# Upfront stereotactic radiosurgery in patients with brain metastases from small cell lung cancer: retrospective analysis of 41 patients

**DOI:** 10.1186/1748-717X-9-152

**Published:** 2014-07-08

**Authors:** Shoji Yomo, Motohiro Hayashi

**Affiliations:** 1 Division of Radiation Oncology, Aizawa Comprehensive Cancer Center, Aizawa Hospital, Matsumoto, Japan; 2 Saitama Gamma Knife Center, San-ai Hospital, Saitama, Japan

**Keywords:** Brain metastases, Small cell lung cancer, Stereotactic radiosurgery, Whole brain radiotherapy

## Abstract

**Background:**

Although the efficacy of prophylactic or therapeutic whole brain radiotherapy (WBRT) for brain metastases (BM) from small cell lung cancer (SCLC) is well established, the role of stereotactic radiosurgery (SRS) has yet to be determined. In the present retrospective analysis, we investigated whether upfront SRS might be an effective treatment option for patients with BM from SCLC.

**Methods:**

We analyzed 41 consecutive patients with a limited number of BM (≤ 10) from SCLC who received SRS as the initial treatment. No prophylactic and therapeutic WBRT was given prior to SRS. The median patient age was 69 years and the median Karnofsky performance status (KPS) score was 90. Repeat SRS was given for new distant lesions detected on follow-up neuroradiological imaging, as necessary. Overall survival, neurological death, and local and distant BM recurrence rates were analyzed. The survival results were tested with three prognostic scoring systems validated for SCLC: Diagnosis-specific graded prognostic assessment (DS-GPA), Radiation therapy oncology group -recursive partitioning analysis and Rades’s survival score.

**Results:**

One- and 2-year overall survival rates were 44% and 17%, respectively. The median survival time was 8.1 months. Survival results replicated the DS-GPA (*P* = 0.022) and Rades’s survival score (*P* = 0.034). On multivariate analysis, patients with high KPS (hazard ratio (HR): 0.308, *P* = 0.009) and post-SRS chemotherapy (HR: 0.324, *P* = 0.016) had better overall survival. In total, 95/121 tumors (79%) in 34 patients (83%) with sufficient radiological follow-up data were evaluated. Six- and 12-month rates of local control failure were 0% and 14%, respectively. Six- and 12-month distant BM rates were 22% and 44%, respectively. Repeat SRS, salvage WBRT and microsurgery were subsequently required in 18, 7 and one patient, respectively. Symptomatic radiation injury developed in two patients and both were treated conservatively.

**Conclusions:**

Our survival analyses with the validated prognostic grading systems suggested upfront SRS for limited BM from SCLC to be a potential treatment option, with patient survival being slightly more than eight months after SRS. Although SRS provided durable local tumor control, repeat treatment was needed in nearly half of patients to achieve control of distant BM.

## Background

Small cell lung cancer (SCLC) is the most aggressive histologic subtype of lung cancer, with a predilection for early metastases [1]. Brain metastases (BM) are a significant threat to quality of life in patients with SCLC [2]. Given that the cumulative incidence of BM from SCLC at 2 years is approximately 50% [1,3], prophylactic cranial irradiation (PCI) combined with systemic chemotherapy, which moderately prolongs overall survival (OS) by reducing the incidence of delayed BM, has historically been recommended as the treatment for this aggressive disease in most patients [4-8].

Stereotactic radiosurgery (SRS) has now emerged as the preferred treatment modality, either alone or in combination with other modalities. Recently, in selected patients, whole brain radiotherapy (WBRT) has been omitted from the initial management for BM with the aim of reducing the potential risk of delayed neurological toxicity [5,9-11]. Given the propensity for dissemination of SCLC, SRS does not appear to be a rational approach to this pathology. To date, there have been only a few, relatively small, studies of SRS for SCLC, which focused mainly on salvage treatment for recurrence after WBRT [12-16]. Thus, the role of upfront focal treatment by means of SRS for BM from SCLC has yet to be determined. We retrospectively investigated the efficacy and limitations of an SRS-oriented strategy for patients with newly diagnosed BM from SCLC.

## Methods

### Patient population

Between January 2009 and May 2013, 42 patients with BM originating from histologically proven primary SCLC underwent Gamma Knife SRS as a part of initial management. Prior WBRT had not been conducted in either a prophylactic or a therapeutic setting. All patients included in the present study had been diagnosed and their primary tumors treated at other hospitals, where the appropriateness of SRS had been determined by the clinical oncologist and the patient. They were then referred to our institution to receive SRS for BM. Patients with up to 10 BM received, in principle, SRS as the initial therapy. When abnormal enhancement of cranial nerves, the ventricular ependymal layer and/or the cortical surface or more than 10 BM were documented by high resolution magnetic resonance (MR) imaging at the time of initial SRS, WBRT was indicated and such patients were excluded from the present study. Surgical resection was recommended for large tumors with a mass effect unresponsive to corticosteroid therapy. In the event of surgery not being feasible due to poor systemic condition or prognosis, 2-session SRS was conducted for carefully selected patients with tumors larger than 10 mL [17]. All patients and/or their relatives were fully informed that upfront SRS remains an unproven strategy in terms of safety and efficacy, and all provided written informed consent. San-ai Hospital Institutional Review Board approved this retrospective clinical study in January 2014. The current retrospective review involves a series of 41 consecutive patients who had not more than 10 BM. The one remaining patient receiving upfront SRS for more than 10 BM, due to refusal to undergo WBRT, was excluded. Thirty-four patients were male and 7 were female. The median age was 69 years (range: 50–85). The median Karnofsky performance status (KPS) score at the time of SRS was 90 (range: 30–100). The median interval between initial diagnosis and SRS was 7.8 months (range: 0–149 months). Five patients had undergone microsurgical resection for large symptomatic BM before SRS. The patient characteristics are summarized in Table 1.

**Table 1:** Summary of clinical data from 41 consecutive patients with BM from SCLC

**Characteristics**	**Values**
Sex (male/female)	34 / 7
Age (years), median (range)	69 (50–85)
KPS, median (range)	90 (30–100)
Active Extra-CNS disease or Extra-CNS metastasis	25 (61%)
DS-GPA 0–1.0/1.5-2.5/3.0-4.0	20 / 15 / 6
RTOG-RPA class I/class II/class III	6 / 25 / 10
Post-SRS chemotherapy	32 (78%)
Time from primary diagnosis to initial SRS (months), median (range)	7.8 (0.3-149)
Cumulative PTV on initial SRS (mL), median (range)	3.8 (0.6-28.2)
No. of intracranial lesions on initial SRS, median (range)	2 (1–10)

BM: brain metastases, SCLC: small cell lung cancer, KPS: Karnofsky performance status, CNS: central nervous system, DS-GPA: diagnosis-specific graded prognostic assessment, RTOG-RPA radiation treatment oncology group recursive partitioning analysis, , SRS: stereotactic radiosurgery, PTV: planning target volume.

### Radiosurgical indications and techniques

SRS was performed using the Leksell G stereotactic frame (Elekta Instruments, Stockholm, Sweden). The frame was placed on the patient’s head under local anesthesia supplemented with mild sedation. 3-D volumetric gadolinium-enhanced T1-weighted magnetic resonance (MR) images, 2 mm in thickness, and T2-weighted MR images and contrast-enhanced computed tomography covering the whole brain were routinely used for dose planning with Leksell Gamma Plan software (Elekta Instruments). Prescribed doses were selected in principle according to the dose protocol of the JLGK 0901 study [18], though a margin of approximately 1 to 2 mm was added to the visible lesion in consideration of the infiltrative nature of SCLC [19]. The technical details of 2-session SRS were previously described in detail [17]. All treatments were performed with the Leksell Gamma Knife Model C or Perfexion.

### Post-SRS management and follow-up evaluation

Clinical follow-up data as well as contrast-enhanced MR images were obtained every one to three months. If metachronous distant metastases were identified, they were managed, in principle, with repeat SRS. When miliary metastases (numerous tiny enhanced lesions) and/or leptomeningeal carcinomatosis was newly documented, WBRT was then indicated. Local control failure was defined as an at least 20% increase in the diameter of the targeted lesions, taking as a reference the pre-SRS diameter, irrespective of whether the lesion was a true recurrence or delayed radiation injury. Delayed radiation injury was differentiated from tumor recurrence using the T1/T2 mismatch method [20] and, in selected cases, ^11^C-methionine positron emission tomography. Additional SRS was possible provided that the volume of the local tumor recurrence was small enough for single-dose SRS. Surgical removal was indicated when neurological signs became refractory to conservative management, with a radiological diagnosis of local tumor progression or radiation necrosis. Any adverse events attributable to SRS procedures were evaluated based on the National Cancer Institute Common Terminology Criteria for Adverse Events (NCI-CTCAE; ver.3.0). Before closing the research database for analysis, the authors updated the follow-up data of patients who had not visited our outpatient department for more than two months. Inquiries about the date and mode of death were made by directly corresponding with the referring physician and/or the family of the deceased patient. Neurological death was defined as death attributable to central nervous system (CNS) metastases including tumor recurrence and carcinomatous meningitis. Events such as pneumonia due to a decline secondary to CNS disease progression were also scored as neurological deaths.

### Statistical analysis

The overall survival (OS) rate was calculated by the Kaplan-Meier product limit method. The neurological and non-neurological death rates were calculated employing Gray’s test [21], where each event was regarded as a competing risk for another event. For the estimation of local control failure rates and distant BM recurrence, Gray’s test was similarly used, with subsequent WBRT and the patient’s death being regarded as competing events, respectively. All of the above analyses were based on the interval from the date of initial SRS treatment until the date of each event. Univariate and multivariate analyses were performed using the log-rank test and the Cox proportional hazards model to investigate prognostic factors for OS. Possible prognostic factors were selected with reference to other SRS series [12-16]. The survival results were tested and compared employing three prognostic scoring systems validated for SCLC: Diagnosis-specific graded prognosis assessment (DS-GPA) [22], Radiation therapy oncology group (RTOG)-recursive partitioning analysis (RPA) [23] and Rades’s survival score [24]. The statistical processing software package “R” version 3.0.1 (The R Foundation for Statistical Computing, Vienna, Austria) was used for all statistical analyses. A *P*-value of < 0.05 was considered to indicate a statistically significant difference.

## Results

**Figure 1:**
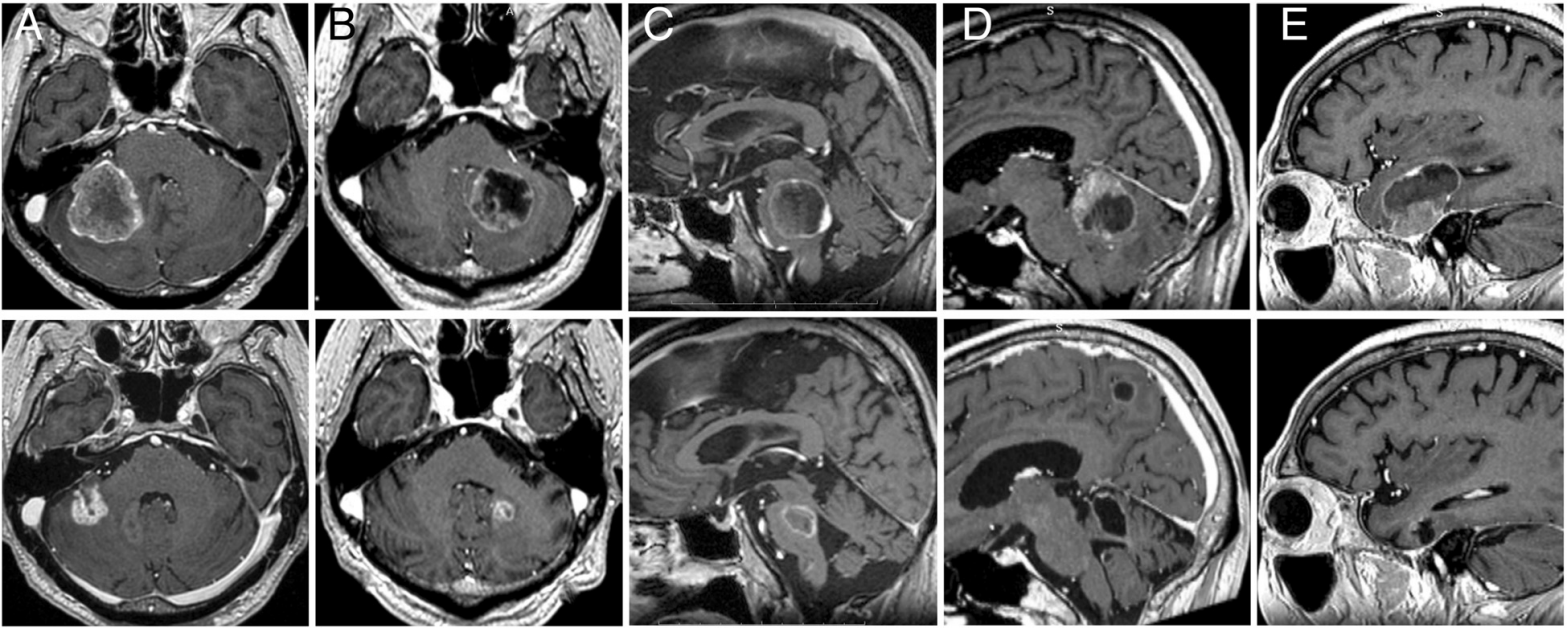
**Contrast-enhanced T1-weighted MR images from 5 patients treated with 2-session SRS.** The upper and lower images were obtained at the time of the initial SRS and the latest follow-up, respectively. A 71-year-old man with a tumor 19.2 mL in volume *(upper),* 3 months post-treatment *(lower)*: **(A)**. A 60-year-old man with a tumor 14.3 mL in volume *(upper)*, 11 months post-treatment *(lower)*: **(B)**. A 64-year-old man with a tumor 15.2 mL in volume *(upper)*, 8 months post-treatment *(lower)*: **(C)**. A 61-year-old man with a tumor 17.0 mL in volume *(upper)*, 14 months post-treatment *(lower)*: **(D)**. An 80-year-old woman with a tumor 22.3 mL in volume *(upper)*, 9 months post-treatment *(lower)*: **(E)**.

Twenty-five patients (61%) had active systemic disease and/or extra-CNS metastases and 32 (78%) received systemic chemotherapy following the initial SRS. In total, 121 tumors were treated at the time of the initial SRS. The median planning target volume (PTV) was 0.6 mL (range: 0.1-19.1 mL). The median number of BM at diagnosis was 2 (range: 1–10 tumors) and the median cumulative PTV was 3.8 mL (range: 0.6-28.2 mL). Prescribed doses ranged from 10 Gy to 22 Gy (median: 20 Gy). Five patients with large tumors were allocated to 2-session SRS (Figure 1).

**Figure 2:**
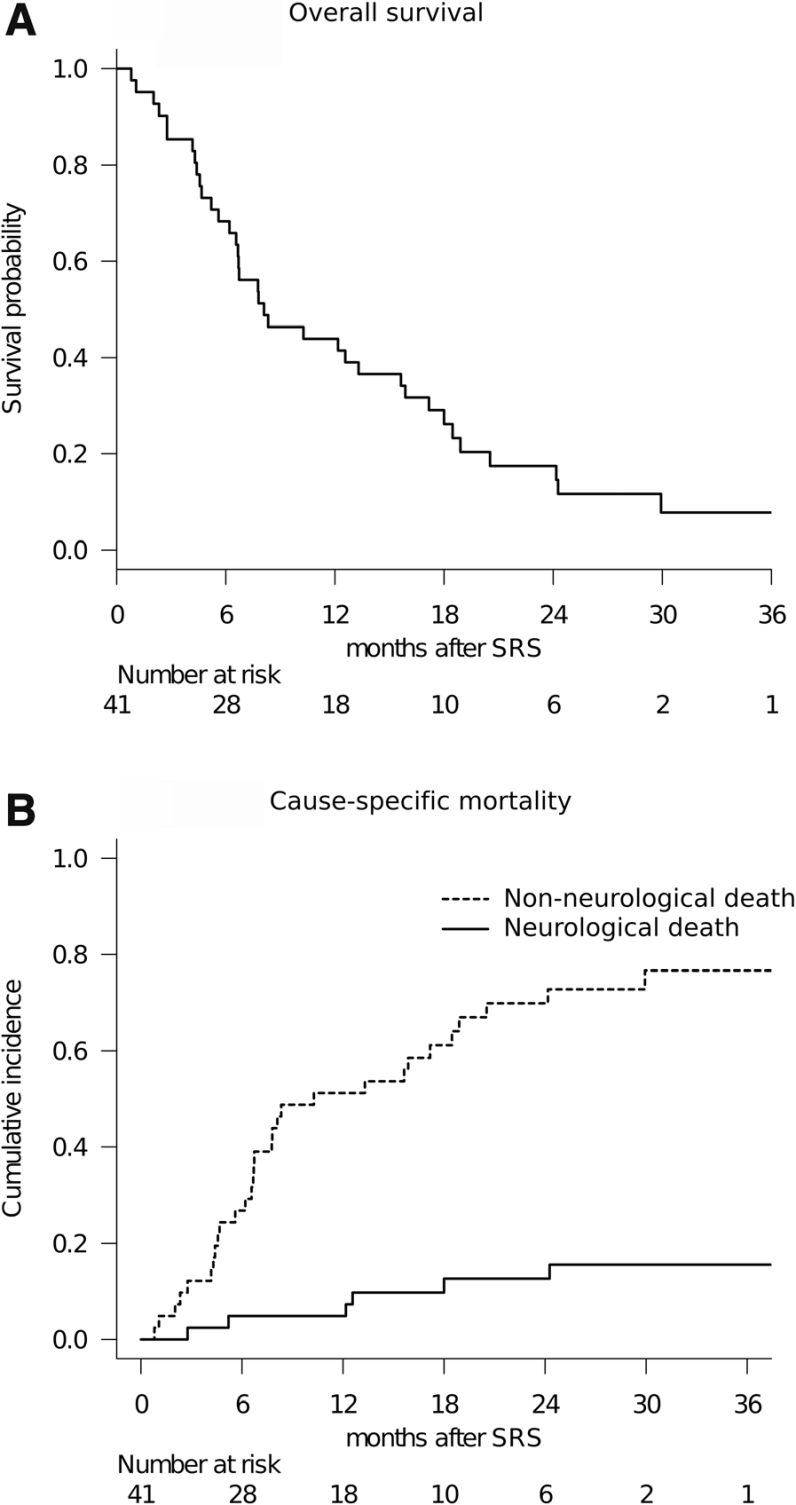
**Overall treatment results of patients with BM from SCLC treated with SRS.** OS after initial SRS: **(A)** The MST was 8.1 months (95% CI: 6.2-15.6). Cumulative incidence of cause-specific mortality: **(B)** The solid and dotted lines represent neurological and non-neurological death rates, respectively.

Full clinical results were available for all 41 patients as none were lost to follow-up. The median follow-up time after SRS was 8.1 months (range: 0.8-37.8). At the time of assessment, 5 patients (12%) were alive and 36 (88%) had died. The causes of death were intracranial local progression in 3 cases, meningeal carcinomatosis in 3 and progression of the primary lesion in 30. The actuarial 1- and 2-year OS rates after SRS were 44% and 17%, respectively (Figure 2a). The median survival time (MST) was 8.1 months (95% confidence interval (CI): 6.2-15.6). Cumulative incidences of 1- and 2-year neurological death after SRS adjusted for competing events (non-neurological death) were 5% and 13%, respectively (Figure 2b). The results of analyses for variables possibly correlating with OS are shown in Table 2. In univariate analysis, high KPS (*P* < 0.001), controlled extra-CNS disease (*P* = 0.005), post-SRS chemotherapy (*P* = 0.009) and having a single BM (*P* = 0.011) influenced OS rates significantly. The proportional hazards model for OS identified high KPS (HR: 0.308, 95% CI: 0.128-0.742, *P* = 0.009) and post-SRS chemotherapy (HR: 0.324, 95% CI: 0.130-0.809, *P* = 0.016) as the only two prognostic factors independently predicting OS rates.

**Table 2:** Statistical analysis of factors predicting patient survival after SRS

**Parameter (No. of patients)**	**OS (months)**	Univariate*	Multivariate**	
	**Median (95% CI)**	***P *** **Value**	***P *** **Value**	**HR (95% CI)**
Age		0.205	0.456	0.758 (0.363-1.58)
65 years or less (17)	12.6 (6.2-20.5)			
More than 65 years (24)	6.7 (4.4-13.3)			
KPS		< 0.001	0.009	0.308 (0.128-0.742)
90 or more (23)	13.3 (6.7-24.2)			
Less than 90 (18)	5.4 (4.3-12.2)			
Extra-CNS disease status		0.005	0.450	0.674 (0.242 − 1.88)
None or inactive (16)	18.0 (6.7-29.9)			
Active (25)	6.7 (4.7-12.6)			
Post-SRS chemotherapy		0.009	0.016	0.324 (0.130-0.809)
Yes (32)	12.4 (6.7-18.0)			
No (9)	5.2 (0.8-8.3)			
Total PTV at initial SRS		0.063	0.672	0.842 (0.381-1.86)
5 mL or less (21)	7.8 (6.2-24.2)			
More than 5 mL (20)	8.2 (2.8-15.6)			
No. of BM		0.011	0.161	0.551 (0.240-1.27)
Single (18)	16.8 (5.2-29.9)			
Multiple (23)	6.7 (4.7-12.2)			

SRS: stereotactic radiosurgery, OS: overall survival, CI: confidence interval, KPS: Karnofsky performance status, CNS: central nervous system, PTV: planning target volume, BM brain metastases.

*Log-rank test.

**Cox proportional hazards model.

**Figure 3:**
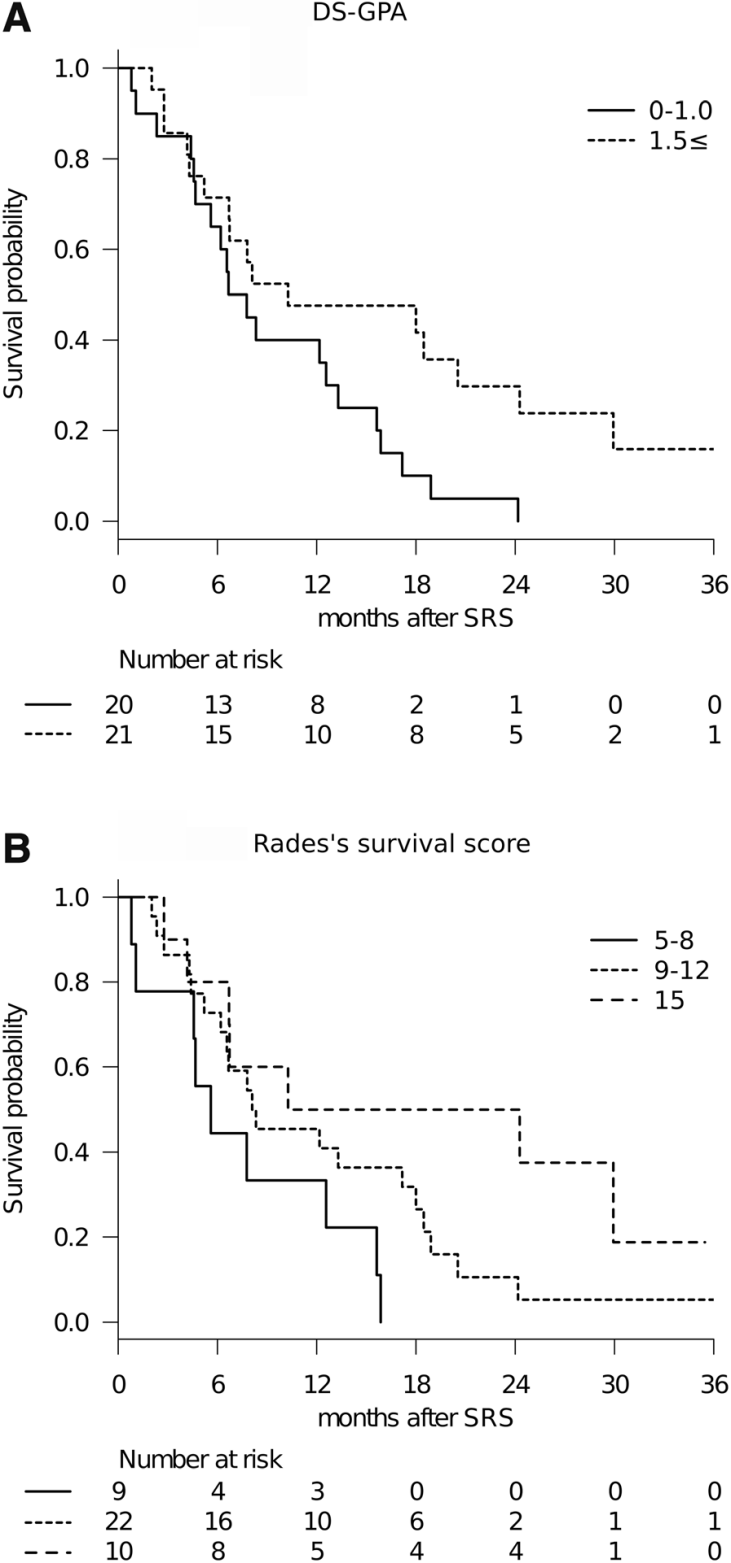
**Survival results stratified with prognostic scoring systems.** Diagnosis-specific graded prognostic assessment (DS-GPA): **(A)**. Rades’s survival score: **(B)**.

**Figure 4:**
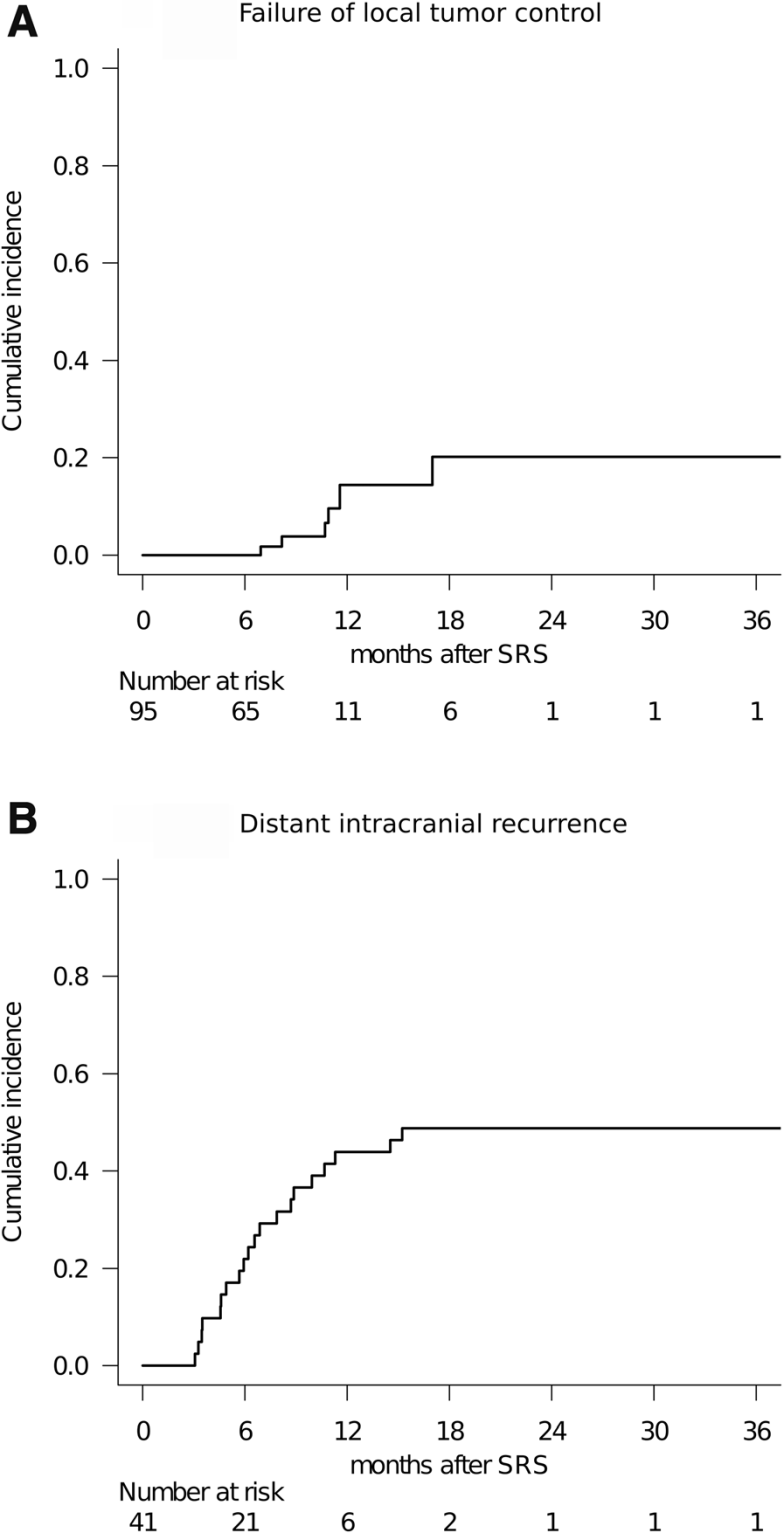
**Cumulative incidence of local and distant brain failure.** The 6- and 12-month local tumor control failure rates were 0% and 14%, respectively: **(A)**. The 6- and 12-month distant intracranial recurrence rates were 22% and 44%, respectively: **(B)**.

The survival results were tested with validated prognostic scoring systems (Table 3, Figure 3). The DS-GPA showed significant differences in MST when patients were dichotomized prior to comparison, due to the small number of patients included: DS-GPA 1.5 ≤: 10.3 months (95% CI: 5.2-24.3), DS-GPA 0–1.0: 7.2 months (95% CI: 4.6-13.3), (*P* = 0.022, log-rank test) (Figure 3a). MSTs by RTOG-RPA class were: class I: 9.2 months (95% CI: 2.8-NA), class II: 8.3 months (95% CI: 6.2-18.5), class III: 6.7 months (95% CI: 0.8-15.6). The MST decreased by class, but the difference was not statistically significant (*P* = 0.188, log-rank test). A survival scoring system specifically for patients with BM from SCLC, as proposed by Rades et al. [24], allowed stratification by 6-month patient survival rates: 15 points: 80% (95% CI: 41–95), 9–12 points: 73% (95% CI: 49–87), 5–8 points: 44% (95% CI: 14–72) (*P* = 0.034, log-rank test) (Figure 3b).Only the 95/121 tumors (79%) in 34 patients (83%) who had sufficient radiological follow-up data were analyzed herein because the other 7 patients died from extra-CNS progression without follow-up MR imaging. Six metastases (6%) were eventually diagnosed as local recurrence or delayed radiation injury at a median time of 10.8 months after SRS (range: 6.9-17.0 months). The 6-month and 1-year actuarial rates of local tumor control failure were 0% and 14%, respectively (Figure 4a). Two-session SRS conducted in five patients achieved a durable tumor volume reduction coupled with symptom relief in all cases with sufficient radiological follow-up (Figure 1), though one of these patients experienced local tumor recurrence in the brainstem which eventually resulted in neurological death (Figure 1c). Twenty of the 41 patients eventually developed distant BM recurrence at a median time of 6.4 months after SRS (range: 3.1-15.2 months). The actuarial 6- and 12-month distant BM recurrence rates were 22% and 44%, respectively (Figure 4b).

**Table 3:** Survival of patients with BM from SCLC: Historical comparison using prognostic classification systems

**Author & year (No. of patients)**	**Present study (41)**	**Sperduto et al. 2010 **[22]**(299)**	**Videtic et al. 2007 **[23]**(154)**	**Rades et al. 2013 **[24]**(86)**
Initial treatment				
SRS	100%	4%	1%	0%
WBRT	0%	83%	84%	100%
Patients with 1–3 BM	68%	61%	NA	28%
Overall MST in months	8.1	4.9	4.9	NA
DS-GPA (MST in months)				
0-1.0	7.2 (20)	2.8		
1.5-2.5	8.1 (15)	5.3		
3.0	24.3 (5)	9.6		
3.5-4.0	10.3 (1)	17.1		
RTOG-RPA (MST in months)				
Class III	6.7 (6)		2.5	
Class II	8.3 (25)		5.3	
Class I	9.2 (10)		8.6	
Rades’ score (6-months survival rate)				
5-8	44% (9)			3%
9-12	73% (22)			41%
15	80% (10)			89%

BM brain metastases, SCLC small cell lung cancer, SRS stereotactic radiosurgery, WBRT whole brain radiotherapy, NA not available, MST median survival time, DS-GPA diagnosis specific-graded prognosis assessment, RTOG-RPA radiation treatment oncology group recursive partitioning analysis.

Eighteen patients (44%) required repeat SRS for remote or local BM recurrence. The total number of SRS sessions ranged up to 5 (median: 1) and the total number of BM treated per patient up to 28 (median: 3). Seven patients (17%) had to undergo salvage WBRT at a median time of 10.8 months after SRS (range: 6.1-22.6 months) necessitated by the subsequent development of miliary BM and/or leptomeningeal dissemination. Microsurgical resection was necessary for local tumor recurrence in one patient at 15 months after SRS.

None of the adverse effects observed in this series exceeded NCI-CTCAE grade 3 toxicity. Two patients developed delayed radiation injury (NCI-CTCAE grade 3), which significantly impacted their quality of life due to neurological deficits. Both required prolonged use of steroids coupled with hyperbaric oxygen therapy and eventually showed clinical and radiological stabilization.

## Discussion

BM continues to be a significant threat to quality of life in patients with lung cancer. The risk of developing BM in SCLC is higher than with other histologies. Seute et al. reported the cumulative risk of BM at 2 years after the diagnosis to be 49% to 65% in SCLC [25]. Because of this high likelihood of BM from SCLC, PCI has long been advocated to reduce or delay BM development [3,4,6]. For newly diagnosed BM from SCLC, WBRT has been shown to provide a moderate extension of life and amelioration of neurological symptoms [22,26,27]. However, survival remains poor in patients with BM, probably because the presence of BM is associated with systemic disease relapse [28].

Out of concern over the potential neurotoxicity associated with WBRT [5,11] seen in long-term survivors, and in the hope that local control and survival can be further improved over the results achieved with WBRT, SRS has recently been used as an alternative in selected patients with a limited number of BM and primary histologies other than SCLC. The delivery of highly focused radiation with a sharp dose fall-off is theoretically expected to reduce delayed neurotoxicity. Despite increasing interest in the use of SRS, a strategy that attempts to omit the use of WBRT in the management of BM from SCLC must be adapted with great caution. The argument that the pathology of SCLC is unsuitable for SRS, because of the disseminated nature of this malignancy, cannot be ignored.

What is the clinical significance of MST being slightly more than eight months? The DS-GPA index validated by Sperduto et al. is one of the most reliable and widely used diagnostic tools for predicting the survival of patients with newly diagnosed BM [22]. The DS-GPA did, in fact, predict survival with statistical significance in the present study as well. In the original DS-GPA study, where the majority of patients (82.6%) received WBRT as the sole treatment, the survival of patients with newly diagnosed BM from SCLC was 4.90 months, which was obviously worse than those for patients with tumors at other primary sites. If confined to DS-GPA scores no more than 1.0, the MST was as short as 2.79 months. Considering that almost half the patients had DS-GPA scores of 1 or less in our cohort, the survival outcomes after SRS appear to be acceptable. When it comes to the RTOG-RPA, only 51 patients (4%) in the original RTOG database had SCLC histology [23]. Videtic et al. validated the RTOG-RPA classes for the prognostic stratification of BM even in the setting of SCLC in their retrospective analysis of 154 patients [27]. In their experience, 130 patients (84%) underwent WBRT alone and the MST was 4.9 months, which coincidentally was equivalent to that of the DS-GPA. Historical comparison showed the MST in our cohort (8.1 months) to be similar to that of RTOG-RPA class I (8.6 months). More recently, Rades et al. proposed a new survival scoring system specifically for patients with BM from SCLC, wherein therapeutic WBRT alone was employed and 6-month survival rates were compared. KPS, number of BM, and extracranial metastasis were associated with survival and were thus included in the score. This scoring system predicted the survival rates in our cohort as well, with the rates in the present study apparently being higher in the lower score classes.

The lack of effective therapies for achieving long-term control of extracranial disease makes the interpretation of intervention studies in BM from SCLC difficult [1]. The results of applying local therapy to the brain are likely to be confounded by the competing high risk of death from extracranial disease. Thus, in the present study, we also conducted a competing risk analysis for appropriate evaluation of the efficacy of such a local treatment and the results suggested that continuing active radiosurgical management might reduce neurological death rates. Although it is impossible to compare the rate of neurological death because previous studies, unfortunately, did not provide information about the mode of death, our experience suggests that reducing the neurological death rate by adequately controlling BM might contribute to prolonging OS.

With regard to prognostic factors, high KPS and post-SRS chemotherapy were associated with improved patient survival in both uni- and multivariate analyses. These results are consistent with those of previous studies focusing mainly on salvage treatment. Identifying prognostic factors for longer survival in patients with BM would be critically important for assigning patients to the optimal treatment modality. This observation suggests that selected subsets of patients can be expected to experience prolonged survival, although the expected survival of patients with BM from SCLC may be limited in the majority of cases.

Some of our patients experienced metachronous recurrence outside the treated area after the initial SRS. In fact, subsequent SRS was needed in nearly half of our patients (18; 44%), mostly because of remote BM recurrence. These patients were successfully managed with minimal toxicity by virtue of early detection of local or remote recurrence. As to making this treatment strategy feasible, meticulous clinical and radiographic post-SRS follow-up is required to monitor for recurrent disease. Only seven of our patients (17%) eventually required salvage WBRT because of miliary metastases or leptomeningeal dissemination, neither of which is treatable with SRS. We strategically withhold WBRT until it is presumably the most efficient treatment option, since patients with BM are living longer than ever and may thus be at risk for late manifestation of leptomeningeal carcinomatosis. This strategy may, however, also be fraught with the hazards of delayed intervention. We must establish a more efficient framework for appropriately assigning patients to either WBRT or SRS, to achieve longer survival as well as less treatment-related toxicity.

The patient inclusion criteria, in terms of the number of BM, are more aggressive than those employed in previous studies because our treatment protocol, in principle, referred to that of the JLGK 0901 study prospectively investigating the results of gamma knife SRS for up to ten BM without upfront WBRT. The criticism that such an upfront SRS strategy has not yet been validated for BM from SCLC and its safety is still of major concern must be acknowledged. Adverse events observed in the present series were rare and toxic severity was NCI-CTCAE grade 3 at a maximum, which we consider to be acceptable given the difficult clinical situations. However, the evidence of clinical safety and efficacy remains insufficient and awaits validation in future studies.

The present results must be interpreted with great caution. Although the treatment results after upfront SRS suggested survival similar to that obtained with therapeutic WBRT in properly stratified populations, a patient selection bias inherent to the retrospective approach is unavoidable. One of the critical issues in the present study is that the reason for PCI having been omitted could not be specified for all cases. In addition, the present study included more patients with smaller numbers of BM (≤3), as compared to the past studies cited (Table 3). It should be appreciated that the current study cannot address the potential role of SRS in comparison to WBRT because this was not a randomized but rather a small retrospective observational study. The survival advantage in previous randomized trials supporting PCI as the standard of care also cannot be ignored. However, recent refinements of the SRS apparatus and imaging techniques as well as advancements in systemic therapy and patient care have the potential to influence the modern management of BM from SCLC. Whether an upfront SRS strategy omitting WBRT is a rational alternative to conventional radiation therapy in patients with BM from SCLC can be elucidated only by a prospective randomized study. We consider the present retrospective study to have been necessary as a means of hypothesis generation for future investigation of such issues. Another criticism, that the study time frame is more recent than those of the previous studies and would thus tend to have better results, can also be raised. However, over the last two decades, the survival of patients with SCLC has improved very little (0.63 days per year), despite numerous attempts to identify more efficacious treatments [29]. When it comes to SCLC patients with BM, OS has remained around 5 months since a multi-institutional European phase II study reported that the MST in SCLC BM patients, with no signs of extracranial tumor involvement, was 4.7 months in 1998 [26]. Another weakness of this study is that the relatively small sample size may have resulted in the dataset being underpowered to assess hypotheses and potential prognostic factors. We plan to accumulate further experience with this treatment strategy, in hopes of drawing more robust conclusions. An additional weakness of this study is that only 83% of patients had radiological follow-up, which could lead to overestimation of the true local and remote BM control rates.

## Conclusion

The OS in patients with no more than 10 BM from SCLC was slightly more than eight months after upfront SRS. Patient survival could be predicted by the DS-GPA and Rades’s prognostic grading systems. The current study results suggest upfront SRS to be a potentially effective and minimally invasive treatment option for selected patients with limited numbers of BM from SCLC. Although SRS provided durable local tumor control, repeat salvage treatment was needed in nearly half of our patients to achieve control of distant BM.

## Abbreviations

WBRT: Whole brain radiotherapy; BM: Brain metastases; SCLC: Small cell lung cancer; SRS: Stereotactic radiosurgery; KPS: Karnofsky performance status; DS-GPA: Diagnosis-specific graded prognostic assessment; HR: Hazard ratio; PCI: Prophylactic cranial irradiation; OS: Overall survival; MR: Magnetic resonance; NCI-CTCAE: National cancer institute common terminology criteria for adverse events; CNS: Central nervous system; RTOG: Radiation therapy oncology group; RPA: Recursive partitioning analysis; PTV: Planning target volume; MST: Median survival time; CI: Confidence interval.

## Competing interests

The authors declare that they have no competing interests.

## Authors’ contributions

SY performed the radiosurgical management of these patients and prepared the manuscript. MH provided critical review of the manuscript for important intellectual content. Both authors have read and approved the final manuscript.
